# The prognostic role of the preoperative platelet to lymphocyte ratio in laryngeal cancer

**DOI:** 10.7150/jca.87397

**Published:** 2023-09-11

**Authors:** Qin Li, Mengqi Chen, Lamei Xiao, Huaqin Zhao

**Affiliations:** 1 Department of Clinical Laboratory, Mianyang Central Hospital, School of Medicine, University of Electronic Science and Technology of China, Mianyang, 621000, Sichuan, China.; 2 The Second People's Hospital of Deyang City, Deyang, 618000, Sichuan, China.

**Keywords:** platelet to lymphocyte ratio, laryngeal cancer, prognosis, survival outcome

## Abstract

**Background:** In recent years, several studies have investigated the relationship between platelet to lymphocyte ratio (PLR) and the prognosis of patients with laryngeal cancer, but the results remain controversial. This study aimed to evaluate the prognostic significance of pretreatment PLR in patients with laryngeal cancer.

**Methods:** Up to July 2023, we searched PubMed, PubMed Central, Web of Science, Embase, CNKI and Wanfang databases to collect relevant articles evaluating the relationship between PLR and the prognosis of laryngeal cancer. The pooled hazard ratio (HR) and 95% confidence interval (CI) were calculated using the random effect-model.

**Results:** A total of 14 included studies involving 3220 patients with laryngeal cancer were included. The combined results suggested that elevated PLR was associated with poorer overall survival (HR = 2.21, 95% CI, 1.67 - 2.93, p < 0.001), progression-free survival (HR = 2.54, 95% CI, 1.76-3.66, p < 0.001), recurrence-free survival (HR = 1.87, 95% CI,1.45 - 2.42, p < 0.001), and disease-free survival (HR = 1.46, 95% CI, 1.08-1.98, p < 0.001). Subgroup analysis further confirmed that pretreatment PLR was an independent predictor of OS in laryngeal cancer patients.

**Conclusion:** Higher pretreatment PLR is strongly related to poor prognosis of laryngeal cancer patients. This indicates that PLR has the potential to serve as a valuable biomarker for predicting the prognosis of laryngeal cancer. However, further validations in large prospective cohorts are necessary to confirm its clinical utility and reliability.

## Introduction

Laryngeal cancer is one of the most common tumors of the respiratory tract, with epidemiological surveys showing approximately 12,400 new cases and 3,800 deaths from laryngeal cancer in the United States in 2022 1. Despite considerable progress in minimally invasive surgery and radiotherapy techniques, the survival rate of laryngeal cancer patients has shown limited improvement in recent years 2. Moreover, even patients who have undergone adjuvant radiotherapy after surgery remain vulnerable to recurrence. Consequently, there is a pressing need to enhance prognostic outcomes by identifying valuable prognostic factors and pinpointing individuals at high risk of recurrence.

Numerous studies have shown the involvement of the inflammatory response in the occurrence, progression, and metastasis of cancer 3. Consequently, there has been a surge of scholarly interest in exploring tumor-associated inflammatory markers. One such marker is the platelet to lymphocyte ratio (PLR), which is calculated as the ratio of absolute platelet count to absolute lymphocyte count. Elevated PLR has been consistently linked to poorer survival outcomes in various cancer patients 4. Specifically concerning laryngeal cancer, several studies have recently examined the association between PLR and patient prognosis 5-7. However, the role of PLR in predicting the prognosis of laryngeal cancer remains a subject of controversy.

In order to address this matter comprehensively, we assembled pertinent publications and conducted a meta-analysis to evaluate the significance of PLR in the prognosis of laryngeal cancer. This study aims to furnish robust medical evidence to inform clinical practice.

## Materials and methods

### Search strategy

We performed the systematic review according to the Preferred Reporting Items for Systematic Reviews and Meta-analyses (PRISRMA) reporting guidelines 8. As of July 2023, we extensively searched multiple databases, including PubMed, PubMed Central, Web of Science, Embase, CNKI, and Wanfang, to identify relevant articles investigating the association between PLR and survival outcomes in laryngeal cancer. A literature search was conducted using the following terms: (“Platelet-to-lymphocyte ratio” or “platelet-lymphocyte ratio” or “platelet to lymphocyte ratio” or “platelet lymphocyte ratio” or “PLR”) AND (“laryngeal neoplasm” or “laryngeal Cancer” or “laryngeal tumor” or “laryngeal carcinoma” or “larynx neoplasm” or “larynx cancer” or “larynx tumor” or “larynx carcinoma”). Furthermore, we performed a manual search for references cited in the selected articles and related studies. This process was executed independently by the two authors, and any disagreements that arose were resolved through negotiation.

### Inclusion and exclusion criteria

The inclusion criteria for the study selection were as follows: (1) The studies needed to be clinical cohort studies, randomized clinical trials, or case-control studies. (2) Studies focused on the relationship between preoperative PLR and prognosis of patients with laryngeal cancer. (3) Studies must report hazard ratios (HR) and their corresponding 95% confidence intervals (CI) for overall survival (OS), progression-free survival (PFS), recurrence-free survival (RFS), and/or disease-free survival (DFS) of patients, along with specific data. (4) Studies should provide the exact PLR cut-off value, which was used to categorize patients into high and low PLR groups.

The criteria for exclusion were as follows: (1) Reviews, letters, comments, conference abstracts and case reports. (2) Repeatedly published studies. (3) Studies where full text was not available or data was missing. (4) Studies that did not provide a preoperative PLR or did not have an exact PLR cut-off value.

### Data extraction

For all eligible studies, the following data were extracted: first author's name, year of publication, study region, study type, TNM stage, treatment strategy, number of patients, Patient's age, follow-up time, cut-off values for PLR, HR for clinical prognostic outcomes and its corresponding 95% CI. The two authors extracted data independently, and the differences were resolved through negotiation.

### Quality assessment

We evaluated the quality of the included studies using the Newcastle-Ottawa Scale (NOS), which consists of three components: selection, comparability, and outcome evaluation 9. Each of the three components was assigned a different score (selection: 0-4, comparability: 0-2, outcome evaluation: 0-3). studies with NOS scores < 5 were classified as low quality, studies with NOS scores between 5 and 6 were considered of moderate quality, and studies with NOS scores ≥ 7 were regarded high quality. Two authors did this work independently and disagreements were resolved by negotiation.

### Statistical analysis

The STATA software (version 14.0) was used for all statistical analyses. Survival outcomes, including OS, PFS, RFS, and DFS, were evaluated using a combined HR and 95% CI. When studies have both univariate and multivariate analysis results, we use the results of the multivariate analysis. The Higgins I-squared statistic and Cochran's Q test were used to assess heterogeneity. I^2^ > 50% or p < 0.1 represented significant heterogeneity. The random-effects model was used for pooled analysis, and subgroup analysis and meta-regression were used to look for potential sources of heterogeneity. To validate the reliability of the pooled results, sensitivity analyses were conducted by iteratively excluding individual studies one at a time. In addition, we assessed publication bias using the Egger's test. p < 0.05 was considered statistically significant.

## Results

### Literature search

**Figure 1:**
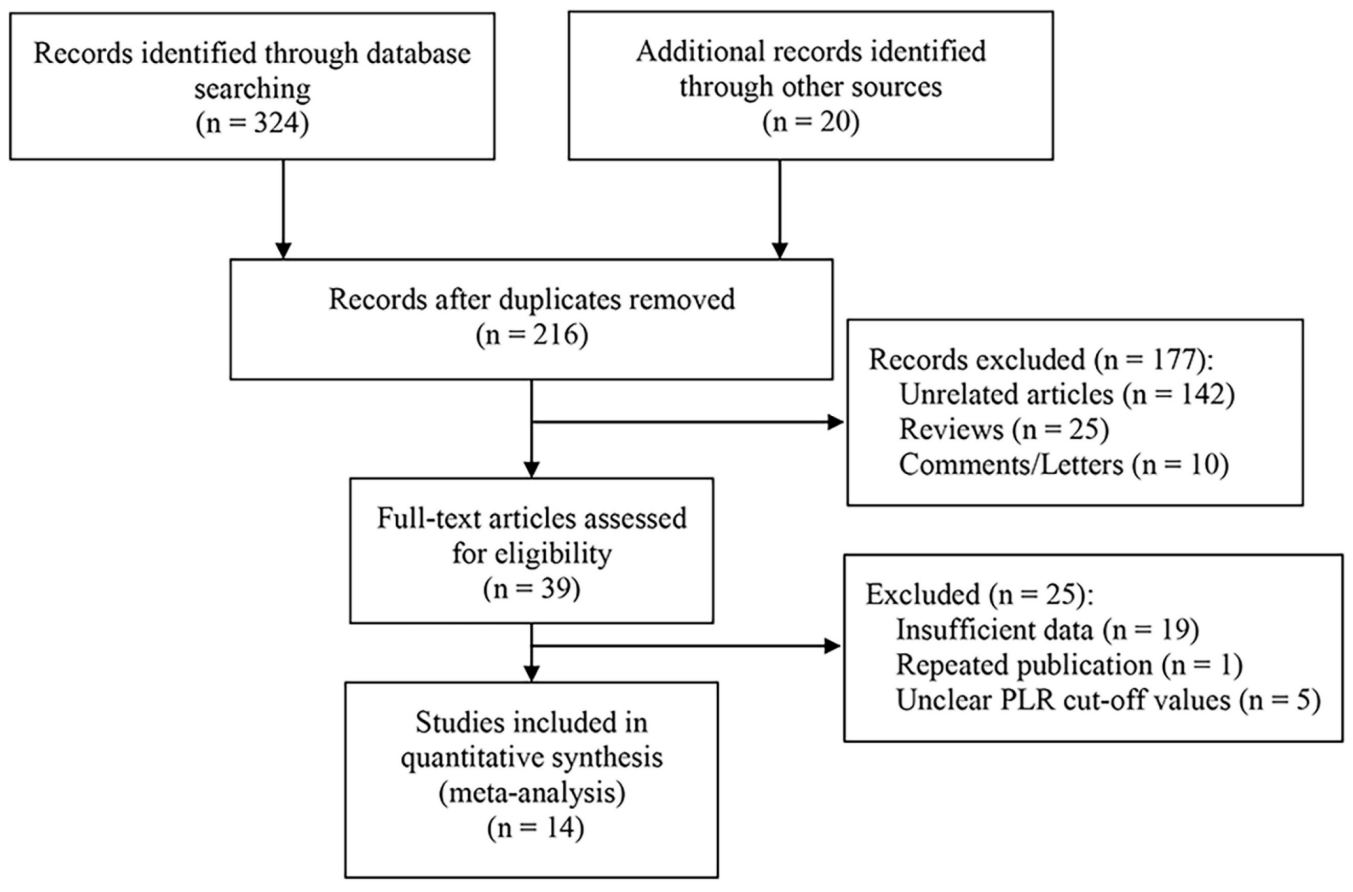
Flow chart for literature screening.

Firstly, 344 studies were identified through a preliminary data search. Of these, 128 were excluded as they were duplicates. After assessing the titles and abstracts, an additional 177 studies were excluded. Subsequently, upon reviewing the full text of the remaining articles, 25 more were excluded. Finally, 14 studies with a total of 3220 patients were deemed eligible for inclusion in this meta-analysis 5-7, 10-20. The literature screening process is visually presented in **Figure 1**.

### Basic characteristics of the included studies

The studies included in this meta-analysis were all retrospective studies. These studies were published between 2016 and 2022, with 11 studies focusing on the relationship between PLR and OS 5-7, 10, 11, 13, 15-20, Four studies focusing on the relationship between PLR and PFS 10, 12, 15, 17, Three studies focusing on the relationship between PLR and RFS 5, 14, 20, Two studies focusing on the relationship between PLR and DFS 6, 13. In addition, the mean/median age of patients included in the studies ranged from 53.2 to 65 years, and the critical values of PLR ranged from 104 to 139.8. In addition, the NOS scores of the included studies were all ≥6 (**Table S1**). The main characteristics of the included studies are shown in **Table 1**.

### The relationship between PLR and OS

**Figure 2:**
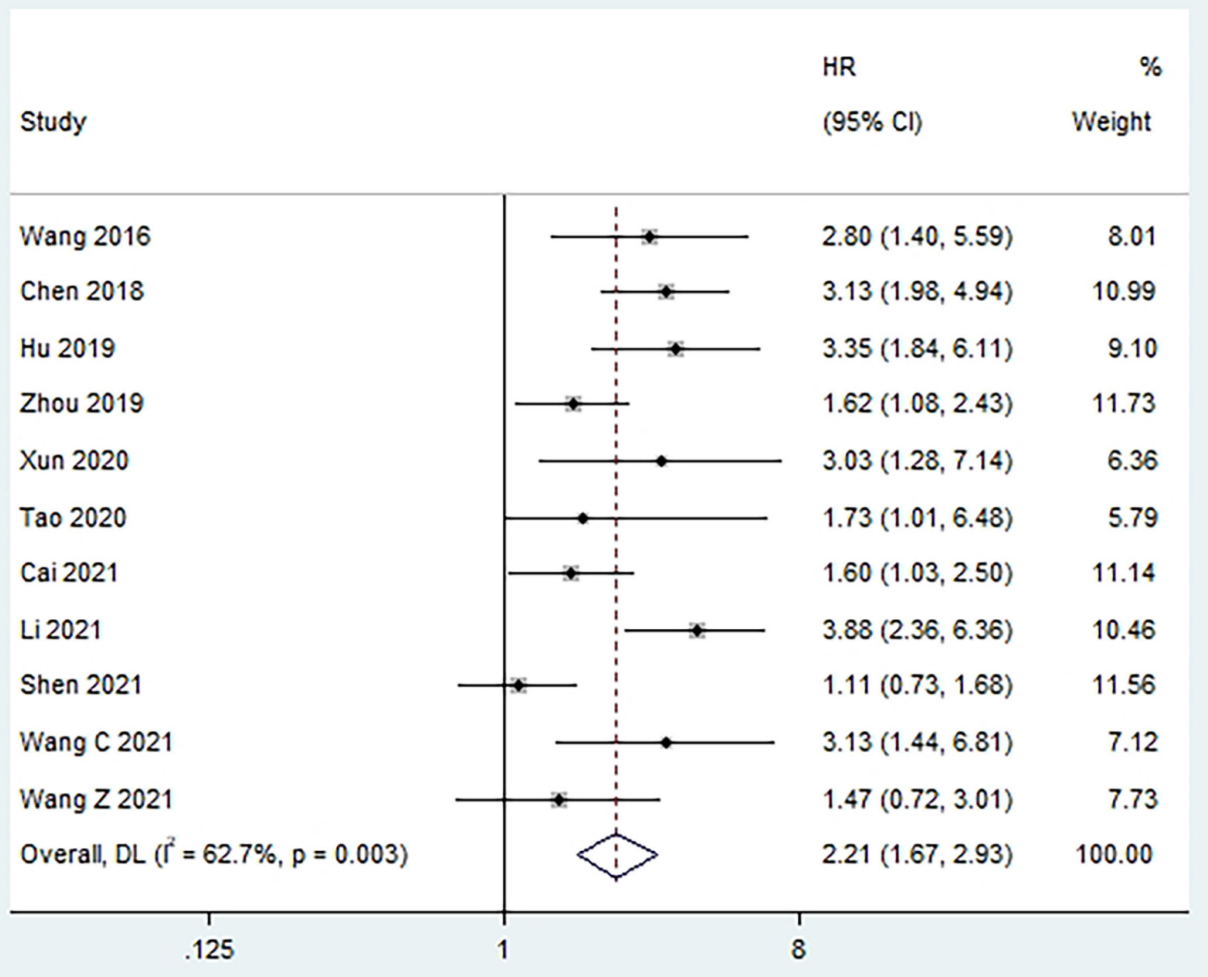
Forest plot of the relationship between high preoperative PLR and OS of patients with laryngeal cancer.

Ten studies reported an association between pretreatment PLR and OS 5-7, 10, 11, 13, 15-18, 20, with tests of heterogeneity indicating moderate heterogeneity between studies (I^2^ = 62.7%, p = 0.003). Results of the pooled analysis showed that elevated PLR was significantly associated with poorer OS in patients (HR = 2.21, 95% CI, 1.67 - 2.93, p < 0.001; **Figure 2**). We performed subgroup analyses based on treatment strategy, PLR cut-off value, sample size, patient age, and analysis methods to explore potential sources of heterogeneity. The results showed that high preoperative PLR was always a risk factor for worse OS in laryngeal cancer patients, regardless of treatment strategy, PLR cut-off value, sample size, Patient's age, and analysis method (**Table 2**). Moreover, meta-regression showed that the OS of patients were not significantly influenced by these factors (p > 0.05,** Table 3**).

### The relationship between PLR and PFS

**Figure 3:**
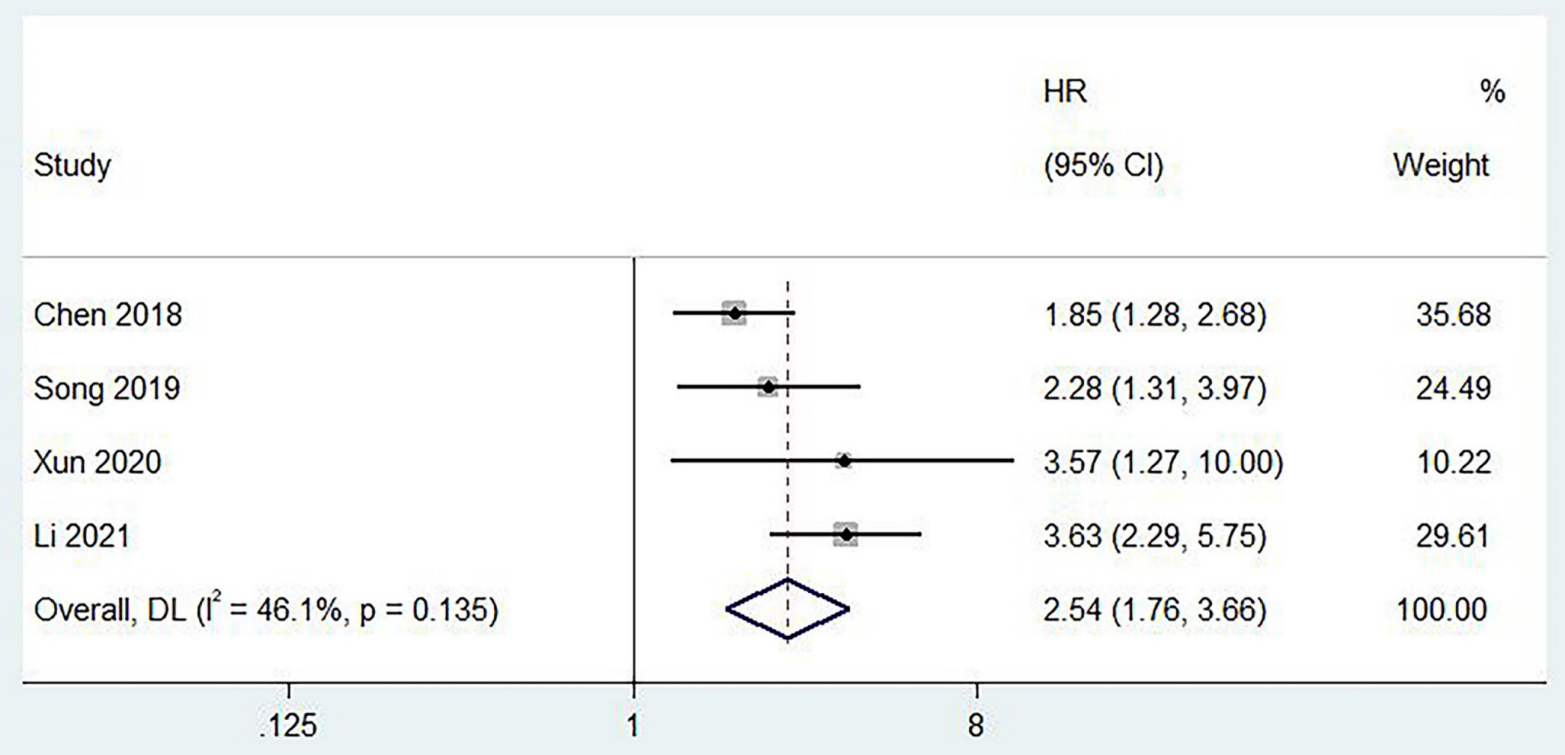
Forest plot of the relationship between high preoperative PLR and PFS of patients with laryngeal cancer.

Four studies reported a correlation between pretreatment PLR and PFS 10, 12, 15, 17, and moderate heterogeneity was found between studies (I^2^ = 46.1%, p = 0.135). The pooled results revealed that a higher preoperative PLR was strongly associated with poorer PFS in patients (HR = 2.54, 95% CI, 1.76 - 3.66, p < 0.001; **Figure 3**).

### The relationship between PLR and RFS/DFS

**Figure 4:**
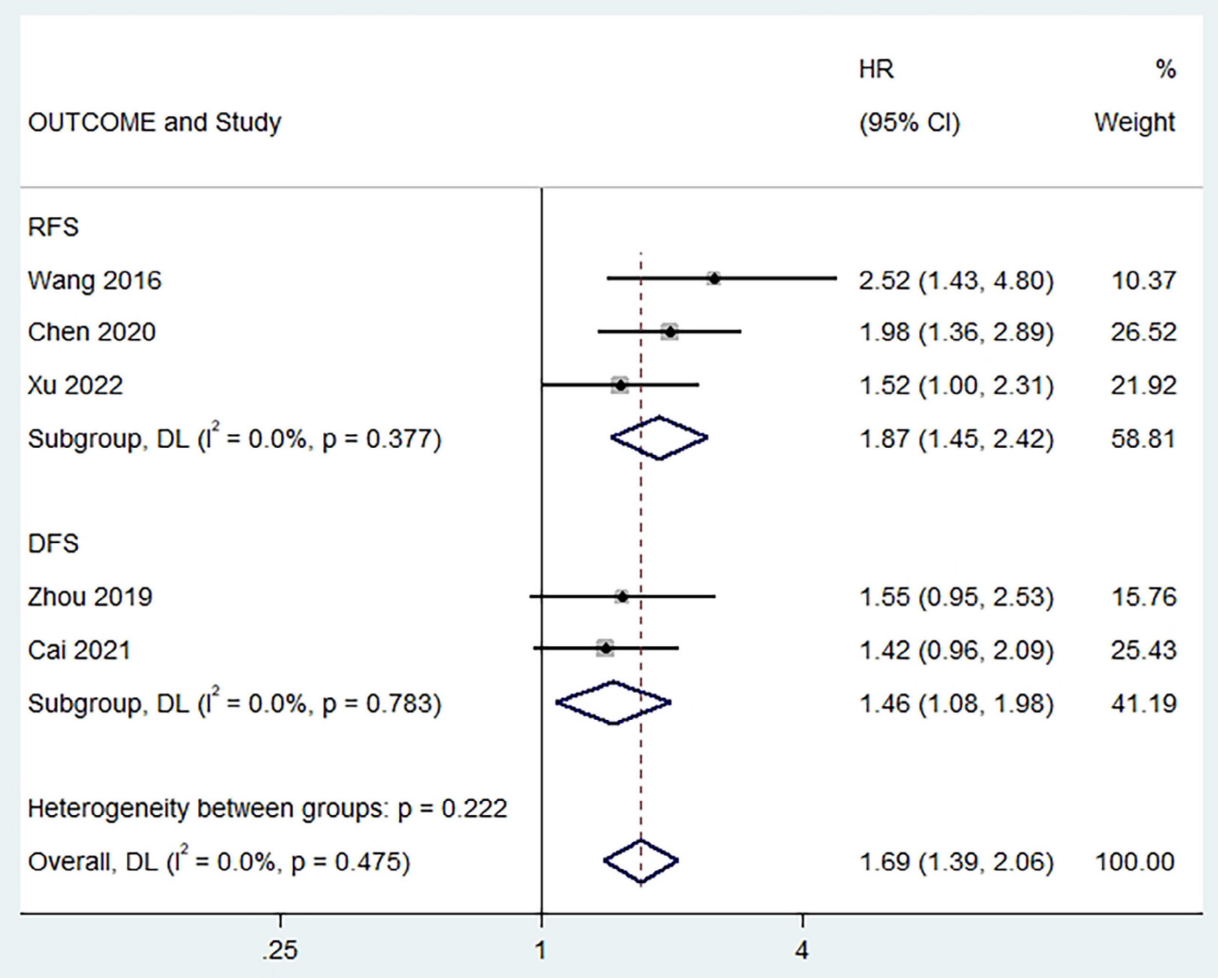
Forest plot of the relationship between high preoperative PLR and RFS/DFS of patients with laryngeal cancer.

Two studies reported a correlation between pretreatment PLR and PFS 6, 13, 19, and no heterogeneity was found between studies (I^2^ = 0%, p = 0.377). The combined results showed that a higher preoperative PLR was significantly associated with worse RFS in patients (HR = 1.87, 95% CI,1.45 - 2.42, p < 0.001; **Figure 4**). In addition, we found the same results in studies exploring the relationship between pretreatment PLR and DFS (HR = 1.46, 95% CI,1.08 - 1.98, p < 0.001, I^2^ = 0%; **Figure 4**).

### Sensitivity analysis

**Figure 5:**
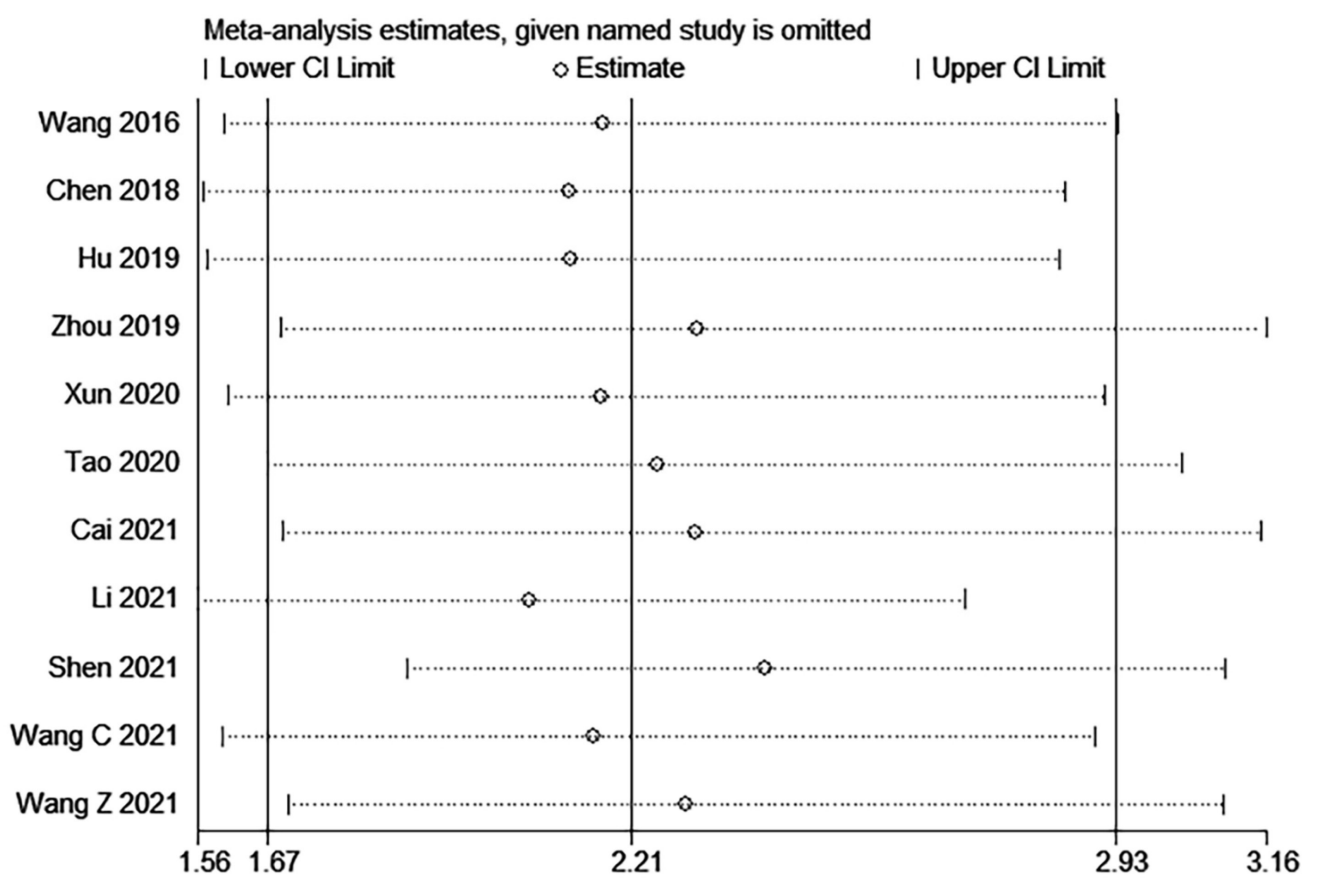
Sensitivity analysis for OS.

The sensitivity analysis of OS indicated that the removal of any single study did not substantially impact the final combined results (**Figure 5**). This finding suggests that the outcomes of this meta-analysis are robust and dependable.

### Publication bias

**Figure 6:**
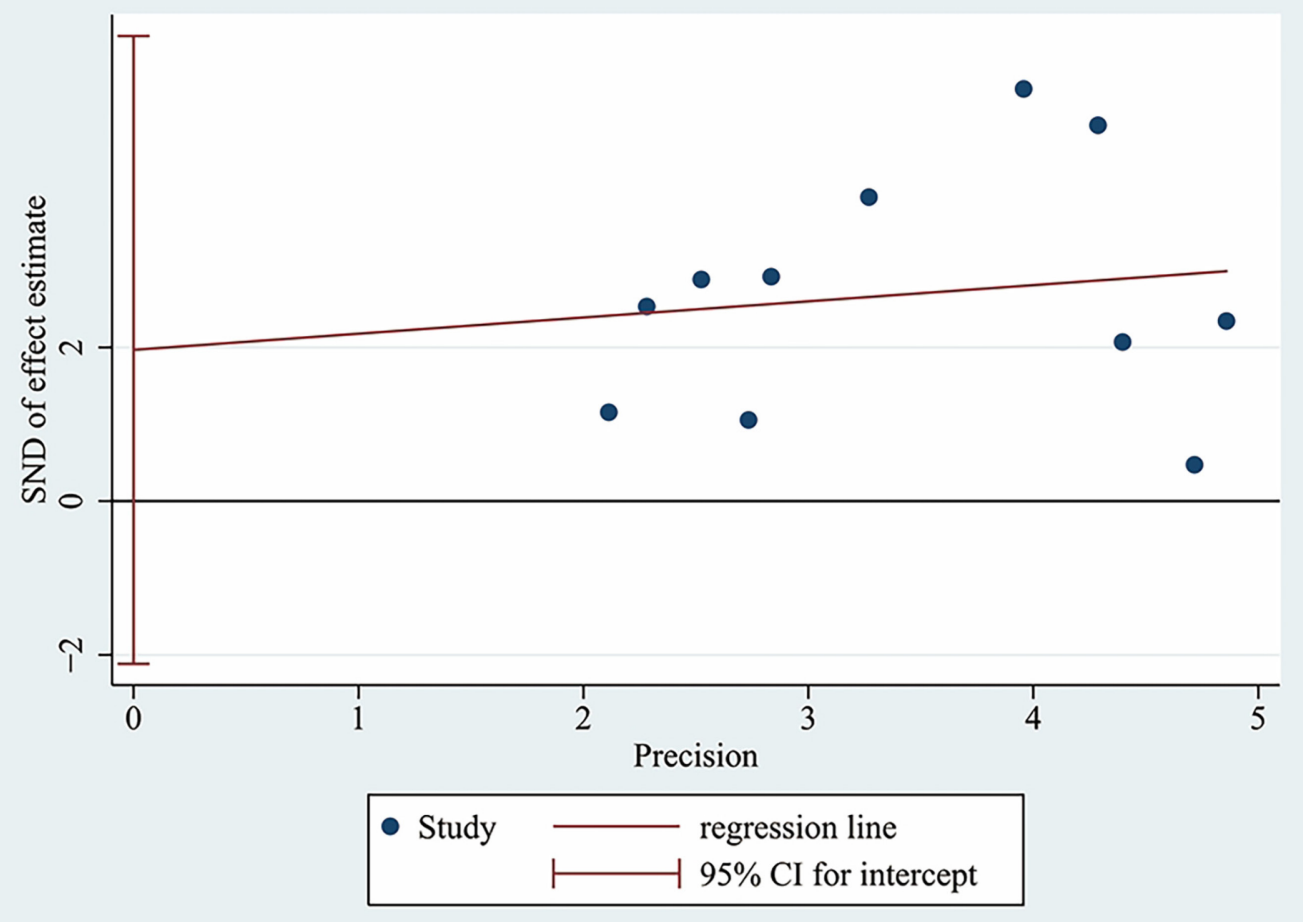
Egger's test for OS.

The Egger's test was used to assess the publication bias of the studies and the results indicated that there was no significant publication bias in this meta-analysis (p = 0.304, **Figure 6**).

## Discussion

Inflammation plays a crucial role in occurrence and progression of tumors. There is growing evidence highlighting the significance of the PLR as a potent prognostic marker for various types of cancers 4. While a systematic review has indicated that high PLR was associated with poor OS 22, but the correlation between PLR and the prognosis of patients with laryngeal cancer remains controversial. Thus, in this study, we conducted a comprehensive review of the existing literature to thoroughly assess the prognostic value of pretreatment PLR in patients with laryngeal cancer.

Compared with the study by u et al. 22, our study has notable strengths. Firstly, we extended the search scope, enabling the inclusion of a broader range of potentially relevant articles. Additionally, we conducted a comprehensive evaluation of multiple tumor prognostic indicators beyond OS, such as DFS, PFS, and DFS, enhancing the reliability of our results. Finally, 5000 patients with laryngeal cancer from 14 studies were included for analysis. The results revealed that patients with high pretreatment PLR experienced poorer OS, PFS, RFS, and DFS. Subgroup analyses further demonstrated a significant association between high PLR and worse OS. Moreover, the findings of the publication bias and sensitivity analyses provided additional confirmation of the reliability and stability of our study. Taken together, our study highlights the potential of pretreatment PLR as an essential prognostic predictor for patients with laryngeal cancer. This information could have valuable implications for clinical decision-making and patient management in the context of laryngeal cancer.

Indeed, while PLR has shown potential as a prognostic biomarker for laryngeal cancer, its specific underlying mechanism remains unclear. As PLR represents the ratio of platelets to lymphocytes, it is reasonable to speculate that the mechanism could be related to the functions of these two blood components. Lymphocytes are a fundamental part of the immune system and can reflect anti-tumor immune response 21. They are responsible for identifying and attacking cancer cells, thereby contributing to tumor surveillance and control. Studies have shown that lymphocytes affect the growth of tumor cells and improve the prognosis of patients with malignant tumors by secreting interferon-γ and tumor necrosis factor-α 23, 24. Therefore, lymphopenia usually indicates a poor prognosis for cancer. In addition, tumour cells can secrete substances such as inflammatory mediators, thrombopoietin and leukaemia inhibitory factor, which promote platelet activation 25-28. The elevated platelets promote tumor angiogenesis by releasing inflammatory mediators and vascular endothelial growth factor^5^. There is evidence that platelets contribute to tumor growth and metastasis and counteract the immune response 25, 28, 29. Platelets were found to promote tumour cell metastasis by activating the TGF-β/Smad and NF-κB pathways to induce epithelial mesenchymal transition in tumour cells 30. Therefore, a higher platelet count might contribute to tumor progression and metastasis, leading to poorer prognosis in patients with laryngeal cancer. Based on this background, the PLR reflects the balance between the tumour inflammatory response and the anti-tumour immune response. An elevated PLR may reflect an increase in platelet-dependent inflammatory response or a decrease in lymphocyte-mediated anti-tumor immune response, forming an immune microenvironment conducive to tumor growth and resulting in a poor prognosis for patients 22.

The strength of our meta-analysis lies in our efforts to exclude confounding factors. Patients may be exposed to corticosteroids or antibiotics after surgery, which may affect platelet and lymphocyte levels. Furthermore, the stress of the procedure may also affect systemic inflammation 31. All studies included in this meta-analysis focused on pretreatment blood samples, excluding the effect of treatment on systemic inflammatory indicators. Notably, all patients in the original study did not receive any therapy before treatment, which eliminate the effect of medication on routine blood test results. Moreover, The results of the test indicated that there was no significant publication bias detected in this meta-analysis. This implies that the findings of our study are less likely to be influenced by potential bias arising from the selective publication of studies with certain results, providing further confidence in the reliability of our results.

Nevertheless, there are still some shortcomings to this study. Firstly, it is essential to recognize that all the studies included in our analysis were retrospective, which might introduce selection bias and affect the generalizability of the results. Secondly, the inconsistency in the cut-off values of PLR among the included studies may have introduced heterogeneity in the analysis and could have influenced the overall findings. Standardizing the cut-off values could enhance the reliability of the results. Thirdly, whereas we included patients who underwent surgery, the various post-operative treatment strategies employed in these studies may have influenced patient survival outcomes. Finally, all the included studies were conducted in Asia, potentially limiting the generalizability of our findings to other populations with different genetic backgrounds and environmental factors. To establish a more comprehensive understanding of the prognostic value of PLR in laryngeal cancer, larger prospective studies conducted in diverse populations are needed. Such studies would facilitate the determination of the optimal threshold for PLR as a prognostic indicator and further inform clinical decision-making.

In conclusion, the existing evidence strongly indicates that elevated preoperative PLR is linked to a worse prognosis in patients with laryngeal cancer. Given that PLR is a readily measurable, simple, and non-invasive marker of subclinical inflammation, it holds promise as a potential prognostic predictor for individuals with laryngeal cancer. In clinical practice, combining information on inflammatory markers, TNM staging, and histological subtypes may enable more accurate prognostic assessments for patients with laryngeal cancer. Such comprehensive evaluations could facilitate personalized treatment strategies and improved patient outcomes in the future. Further research and validation in larger prospective studies are warranted to solidify the prognostic utility of PLR in laryngeal cancer management.

## Supplementary Material

Supplementary table.

## Author Contributions

Conceptualization: Qin Li.

Data curation and analysis: Mengqi Chen, Lamei Xiao, Huaqin Zhao.

Literature assessments: Mengqi Chen, Lamei Xiao.

Visualization: Mengqi Chen, Lamei Xiao, Huaqin Zhao, Qin Li

Article writing and editing: Mengqi Chen, Qin Li.

## Figures and Tables

**Table 1:** Baseline characteristics of include studies and quality assessment.

Authors (year)	Region	Study design	TNM stage	Treatment	Number of patients	Age (years)	Cut-off value	Analysis method	Outcomes	Follow-up (months)	NOS score
Wang 2016^5^	China	Retrospective	I-IV	Multimodality therapy	120	Mean 60.6	112	Multivariate	OS, RFS	40.1, 32.7	8
Chen 2018^10^	China	Retrospective	I-IV	Multimodality therapy	361	Median 60	114	Univariate	OS, PFS	47	7
Hu 2019^11^	China	Retrospective	-	Surgery	286	Median 60	149.9	Multivariate	OS	60	8
Song 2019^12^	China	Retrospective	I-IV	Surgery	137	Median 62	109.5	Univariate	PFS	51	6
Zhou 2019^13^	China	Retrospective	I-IV	Multimodality therapy	232	Median 63	116	Multivariate	OS, DFS	27.3	8
Chen 2020^14^	China	Retrospective	I-IV	Surgery	473	Median 63	104	Multivariate	RFS	46	6
Xun 2020^15^	China	Retrospective	I-IV	Surgery	151	Median 65	106	Multivariate	OS, PFS	60	8
Tao 2020^16^	China	Retrospective	I-III	Surgery	170	Median 54.7	114	Multivariate	OS	60	8
Cai 2021^6^	China	Retrospective	I-IV	Surgery	203	Median 60	110.9	Univariate	OS, DFS	-	7
Li 2021^17^	China	Retrospective	I-III	Multimodality therapy	147	Median 60	117.4	Univariate	OS, PFS	54.2	7
Shen 2021^18^	China	Retrospective	I-IV	Multimodality therapy	338	Median 63	122.9	Multivariate	OS	-	8
Wang 2021^19^	China	Retrospective	-	Surgery	107	Mean 53.2	139.8	Multivariate	OS	60	8
Wang 2021^7^	China	Retrospective	I-IV	Surgery	124	Mean 64.3	137	Multivariate	OS	47	7
Xu 2022^20^	China	Retrospective	I-IV	Multimodality therapy	371	Median 64	105.1	Multivariate	RFS	60	7

Abbreviations: OS: overall survival; PFS: progression-free survival; RFS: recurrence-free survival; DFS: disease-free survival.

**Table 2:** Subgroup analyses for OS.

Variables	Variable	No. of studies	Model	HR (95% CI)	P	Heterogeneity	
						I^2^ (%)	P
OS	All	10	Random	2.41 (1.88, 3.10)	< 0.001	43.9	0.066
Treatment strategy	Surgery Multimodality therapy	6 5	Random Random	2.18 (1.58, 3.00) 2.23 (1.38, 3.60)	< 0.001 < 0.001	24.1 80.2	0.253 0.000
Cut-off value	< 116 ≥ 116	5 6	Random Random	2.34 (1.70, 3.21) 2.13 (1.37, 3.33)	< 0.001 < 0.001	25.1 75.6	0.254 0.003
Sample size	< 170 ≥ 170	5 6	Random Random	2.85 (2.04, 3.98) 2.21 (1.67, 2.93)	< 0.001 < 0.001	17.1 67.9	0.306 0.008
Age	< 62 ≥ 62	7 4	Random Random	2.72 (2.06, 3.58) 1.52 (1.08, 2.14)	< 0.001 0.018	35.2 36.8	0.159 0.191
Analysis method	Multivariate Univariate	8 3	Random Random	2.01 (1.45, 2.78) 2.67 (1.58, 4.52)	< 0.001 < 0.001	53.0 73.9	0.037 0.022

Abbreviations: OS: overall survival; HR: hazard ratio; CI: confidence interval.

**Table 3:** Meta-regression for OS.

Variables	Standardized β coefficient	P value	95% CI
Treatment strategy	0.003	0.992	(-0.668, 0.674)
Cut-off value	0.004	0.763	(-0.022, 0.029)
Sample size	-0.001	0.542	(-0.005, 0.003)
Age	-0.051	0.278	(-0.150, 0.049)
Analysis method	0.279	0.376	(-0.398, 0.956)

Abbreviations: CI: confidence interval.

## Abbreviations

PLR: platelet to lymphocyte ratio
PRISRMA: Preferred Reporting Items for Systematic Reviews and Meta-analyses
HR: hazard ratio
CI: confidence interval
OS: overall survival
PFS: progression-free survival
RFS: recurrence-free survival
DFS: disease-free survival
