# Supplementary material for: The prognostic role of the preoperative platelet to lymphocyte ratio in laryngeal cancer
Source: J Cancer. 2023 Sep 11;14(16):2990–7. doi: 10.7150/jca.87397 (PMC10583586; doi:10.7150/jca.87397)
Supplement: Supplementary file 1 — Supplementary table. [file jcav14p2990s1.pdf]

Supplementary tabe 1. Quality evaluation of the eligible studies with Newcastle–Ottawa scale.

	Selection				Comparability		Outcome		
Study	Representativeness	Selection of non-exposed	Ascertainment of exposure	Outcome not present at start	Comparability on most important factors	Comparability on other risk factors	Assessment of outcome	Long enough follow-up (median≥1 year)	Adequacy (completeness) of follow-up
Wang 2016	*	*	*	*	*	-	*	*	*
Chen 2018	*	*	*	*	-	-	*	*	*
Hu 2019	*	*	*	*	*	-	*	*	*
Song 2019	*	*	*	*	-	-	-	*	*
Zhou 2019	*	*	*	*	-	-	*	*	*
Chen 2020	*	*	*	*	-	-	-	*	*
Xun 2020	*	*	*	*	*	-	*	*	*
Tao 2020	*	*	*	*	*	-	*	*	*
Cai 2021	*	*	*	*	-	-	*	*	*
Li 2021	*	*	*	*	-	-	*	*	*
Shen 2021	*	*	*	*	*	-	*	*	*
Wang C 2021	*	*	*	*	*	-	*	*	*
Wang Z 2021	*	*	*	*	-	-	*	*	*
Xu 2022	*	*	*	*	-	-	*	*	*

*indicates criterion met; - indicates significant of criterion not met.
